# *TYR* as a multifunctional reporter gene regulated by the Tet-on system for multimodality imaging: an *in vitro* study

**DOI:** 10.1038/srep15502

**Published:** 2015-10-20

**Authors:** Hongyan Feng, Xiaotian Xia, Chongjiao Li, Yiling Song, Chunxia Qin, Yongxue Zhang, Xiaoli Lan

**Affiliations:** 1 Department of Nuclear Medicine, Union Hospital, Tongji Medical College, Huazhong University of Science and Technology; Hubei Key Laboratory of Molecular Imaging, Wuhan 430022, China

## Abstract

The human tyrosinase gene *TYR* is a multifunctional reporter gene with potential use in photoacoustic imaging (PAI), positron emission tomography (PET), and magnetic resonance imaging (MRI). We sought to establish and evaluate a reporter gene system using *TYR* under the control of the Tet-on gene expression system (gene expression induced by doxycycline [Dox]) as a multimodality imaging agent. We transfected *TYR* into human breast cancer cells (MDA-MB-231), naming the resulting cell line 231-TYR. Using non-transfected MDA-MB-231 cells as a control, we verified successful expression of *TYR* by 231-TYR after incubation with Dox using western blot, cellular tyrosinase activity, Masson-Fontana silver staining, and a cell immunofluorescence study, while the control cells and 231-TYR cells without Dox exposure revealed no *TYR* expression. Detected by its absorbance at 405 nm, increasing concentrations of melanin correlated positively with Dox concentration and incubation time. *TYR* expression by Dox-induced transfected cells shortened MRI T1 and T2 relaxation times. Photoacoustic signals were easily detected in these cells. ^18^F-5-fluoro-N-(2-[diethylamino]ethyl)picolinamide (^18^F-5-FPN), which targets melanin, quickly accumulated in Dox-induced 231-TYR cells. These show that *TYR* induction of melanin production is regulated by the Tet-on system, and TYR-containing indicator cells may have utility in multimodality imaging.

Molecular imaging has shown promise for non-invasive *in vivo* visualisation of cellular processes. In addition to the commonly used magnetic resonance imaging (MRI), positron emission-computed tomography (PET) and single photon emission-computed tomography (SPECT); optical bioluminescence, optical fluorescence, and photoacoustic imaging (PAI) are undergoing extensive investigation as potential modalities to diagnose and evaluate disease. Each imaging modality has its own strengths and weaknesses. Radionuclide-based imaging techniques (PET and SPECT) are highly sensitive, and PET is quantitatively robust, but they have relatively poor spatial resolution. MRI provides high-resolution images but suffers from low sensitivity. Optical imaging is limited by shallow tissue penetration^1^. Multimodality imaging usually combines two modalities to provide both structural and functional information. Examples include PET/CT, PET/MRI, and SPECT/CT. Research on the use of PET, SPECT, and MRI with immunofluorescence and photoacoustic imaging is ongoing^2^.

The design of molecular probes keeps direct and indirect imaging strategies in mind. Direct imaging images a labelled probe bound to a target, usually a protein. An example is the functional superparamagnetic iron oxide nanoparticles used to construct multifunctional nanostructures for PET/MRI or PET/near-infrared fluorescence (NIRF)/MRI^3^,^4^,^5^. This approach requires specific probes for each modality, and the multiple conjugations may affect the specificity of binding and imaging^6^. Indirect imaging is based on reporter genes, using a probe that specifically binds to the gene product. Commonly used reporter gene products include the thymidine kinase produced by herpes simplex virus type 1 (HSV1-tk) and the sodium iodide symporter (NIS) labelled with radiopharmaceuticals^7^,^8^,^9^,^10^, green fluorescent protein (GFP) and firefly luciferase (Fluc)^11^,^12^, used in fluorescence imaging, and ferritin and tyrosinase^13^,^14^, used in MRI. The indirect strategy often needs to fuse two, three, or even more reporter genes into cells. In our previous experiments, a triple-fused reporter gene (*HSV1-tk, GFP and Fluc*) was prepared for PET, fluorescence and bioluminescence imaging^11^. Gene fusion processes are difficult. Linkers, the distance between reporter genes, and the orientation of each reporter gene are the key factors. This has inspired a search for simpler probes.

Human tyrosinase (*TYR*), a key enzyme, catalyses the three most important steps in melanin production, which include oxidation of tyrosine to dopamine (DOPA), DOPA to dopaquinone, and 5, 6-dihydroxyindile to 5, 6-indolequinone^15^. Melanin production rate and yield correlate positively with *TYR* expression and activity^16^. After transduction of *TYR* into cells and encoding an active tyrosinase, melanin synthesis is activated. The advantage of melanin is its multiple properties that can be imaged with different modalities. Its wide absorption spectrum from the ultraviolet to near infrared enables its use in photoacoustic imaging^17^,^18^. Its affinity to iron can be as high as 16% of its own weight^19^. Ionised iron has high signal intensity on MRI T1-weighted images (T1WI), the intensity increasing with increasing ion concentration^14^. In addition, some studies have found that benzamide and its analogues specifically bind to melanin. Several radiopharmaceuticals, ^125^I-BZA, and ^123/131^I-IBZA (for SPECT imaging) have been developed for the diagnosis of melanoma^20^,^21^. Based on the same principle, some PET probes, such as (N-[2-(diethylamino) ethy1]-6-^18^F-fluoropicolinamide) (^18^F-MEL050), have demonstrated high and specific binding to melanin both *in vitro* and *in vivo*^22^. Another positron probe, ^18^F-5-fluoro-N-(2-[diethylamino]ethyl)picolinamide (^18^F-5-FPN), prepared by our group, has been shown to specifically target melanin *in vitro* and *in vivo* with high retention, affinity and favourable pharmacokinetics^23^. Potentially, using *TYR*, as a reporter gene, one could perform PAI, MRI, and PET or SPECT imaging. Previous studies have demonstrated that *TYR* can be used as a multifunctional reporter gene for PAI/MRI or PAI/MRI/PET imaging both *in vitro* and *in vivo*^24^,^25^.

In gene therapy and related gene studies^26^, it has been demonstrated that the timing and degree of gene expression with an activator substance is much better than the sustained expression of a gene product, as the sustained expression of exogenous genes or proteins may result in some unexpected adverse effects^27^. Since the advent of the Tet-off and Tet-on gene expression systems^28^,^29^, both have been widely used in various prokaryotic and eukaryotic models^30^,^31^. *TYR*, to act as a reporter gene, needs to be transfected and integrated into cells, and the Tet-on tetracycline gene induction system is widely used for inducible expression, as it can effectively control gene expression *in vivo* and *in vitro* using doxycycline (Dox) as the activator^27^,^32^.

We integrated a third-generation tetracycline-inducible gene expression system (Tet-On 3G^®^, Clontech™, TakaraBio, Otsu, Shiga, Japan) with *TYR* to establish a new reporter gene system (Fig. 1). The system was evaluated *in vitro* under the control of Dox for providing the feasibility of multimodality imaging.

## Results

### Identification of tyrosine expression in different groups after Lenti-X tet-on 3G-TYR transduction

**Figure 2:**
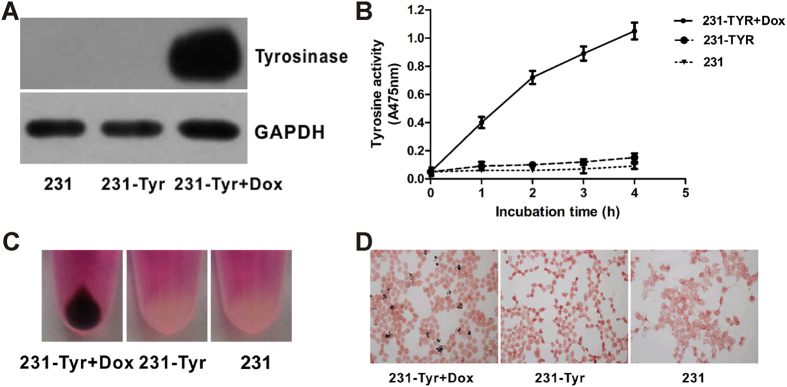
Evaluation of the expression of *TYR* reporter *in vitro*. (**A**) Western blot assay of tyrosinase expression in 231-TYR+Dox, 231-TYR and 231 cells. GAPDH was used as the control. (**B**) Time-response tyrosinase activity curves in three groups. (**C**) Photos of the cell pellets from 231-TYR + Dox, 231-TYR and 231 cells. (**D**) Masson-Fontana silver staining in 231-TYR + Dox, 231-TYR and 231 cells.

We successfully constructed the lentiviral vector Lenti-X Tet-On 3G-TYR, and selected a stable breast cancer cell line expressing *TYR* using puromycin. To measure the expression of the *TYR* gene in 231-TYR + Dox, 231-TYR and 231 cells, western blot was performed (Fig. 2A). We found that *TYR* was only successfully expressed in 231-TYR cells treated with Dox (231-TYR + Dox) and not in the control cells (231-TYR and 231 cells). Cellular tyrosinase activity was also assessed by measuring the amount of dopachrome. Figure 2B shows that the amount of dopachrome in 231-TYR + Dox cells increased over time, while no dopachrome was found in the control groups exposed to Dox. *TYR* activity in 231-TYR + Dox cells was significantly higher than that in the control cells (*P *< 0.05 for all time points). The 231-TYR + Dox, 231-TYR, and 231 cells were collected, and the melanin expression was estimated by visual inspection (Fig. 2C). An obvious black colour was visible in the 231-TYR + Dox cells, while the other cells just showed the colour of the culture medium. Melanin was also identified by Masson–Fontana silver staining, with coarse black particles only found in the 231-TYR + Dox cells (Fig. 2D).

### Results of cell immunofluorescence studies

**Figure 3:**
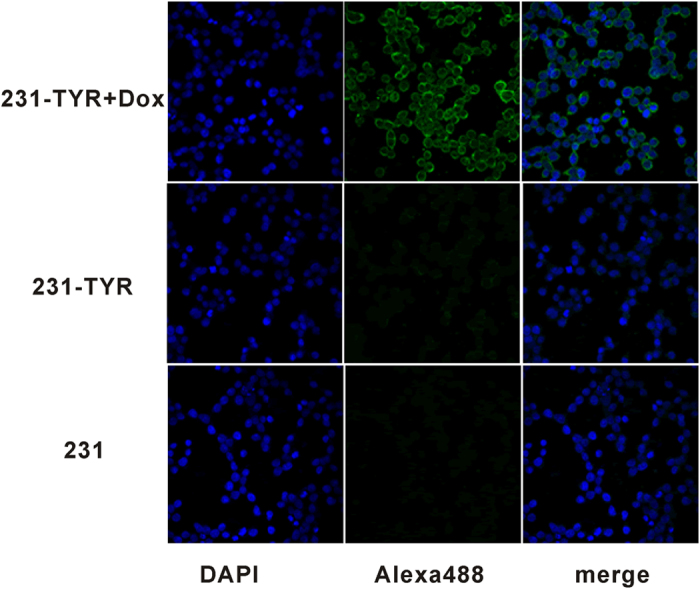
Immunofluorescent staining of TYR in 231-TYR + Dox, 231-TYR and 231 cells. These cells were labelled for immunofluorescence with a TYR-specific monoclonal antibody (green) and their nuclei were counterstained with 4–6-diamidino-2-phenylindole (DAPI) (blue). Representative images (×400) are shown.

To further assess the expression of *TYR*, we performed immunofluorescence experiments. The immunofluorescence results in Fig. 3 demonstrate that *TYR* products were expressed by the 231-TYR + Dox cells, and not by the control cells.

### Dox regulation of melanin production

**Figure 4:**
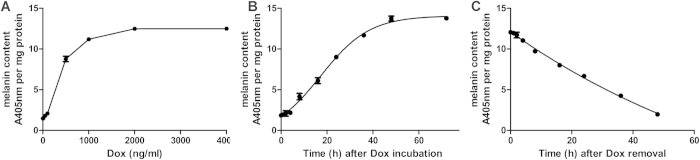
(**A**) Different concentrations of Dox regulate *TYR* expression in 231-TYR cell; (**B**) The incubation time of Dox affect the melanin production in 231-TYR cells; (**C**) the change of melanin content in 231-TYR cells after the withdrawal of Dox at different time.

We quantified the effect of Dox-induced *TYR* expression from the dosage and the duration of exposure to Dox in 231-TYR + Dox cells. As shown in Fig. 4A, the concentration of Dox and melanin yield was positively correlated, melanin production peaking at a concentration of Dox of 2000 ng/mL. Figure 4B displays the Dox-induced melanin yield in 231-TYR cells related to the length of time of Dox incubation, the melanin yield gradually increasing from 4 to 48 h, peaking at 48 h. Melanin began to decrease 4 h after the withdrawal of Dox and returned to normal levels at about 48 h (Fig. 4C). This suggests that Dox should be withdrawn in advance if we want to stop the effect of the reporter gene.

### Cell MRI

**Figure 5:**
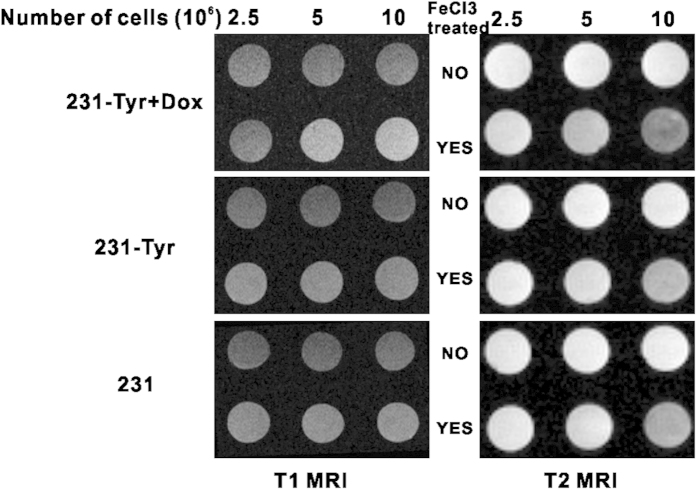
(**A**) T1 MRI (left) and T2 MRI (right) images of three concentrations of 231-TYR + Dox, 231-TYR and 231 cells pre-treated without (top row) or with (bottom row) FeCl_3_.

Different cell concentrations were used to study the sensitivity of MRI for detection of melanin (Fig. 5). We found that 231-TYR + Dox cells cultured with FeCl_3_-enriched medium displayed a much higher signal on T1-weighted images (T1WI), compared with 231-TYR and 231 cells (Fig. 5, left). The T1 relaxation times in msec of 231-TYR + Dox cells with the maximum concentration in the sample with and without FeCl_3_ were 1216.13 and 2470.91 msec, respectively, indication shortening of the T1 relaxation time by 50.78%. We also found that 231-TYR + Dox cells cultured in FeCl_3_-enriched medium displayed much lower signals on T2-weighted images (T2WI), compared with 231-TYR and 231cells (Fig. 5, right). T2 signal decreased with increasing number of 231-TYR + Dox cells. The T2 relaxation times in msec of 231-TYR + Dox cells with the maximum concentration in the sample with and without FeCl_3_ were 29.58 and 84.76 msec, respectively. The iron shortened the T2 relaxation time by 65.1%. The three cell lines cultured in medium without FeCl_3_-enrichment did not produce detectable T1 or T2-weighted signal, and the signals of the control cells with FeCl_3_ treatment only slightly increased and decreased T1 and T2 relaxation times, respectively.

### Cell PAI

**Figure 6:**
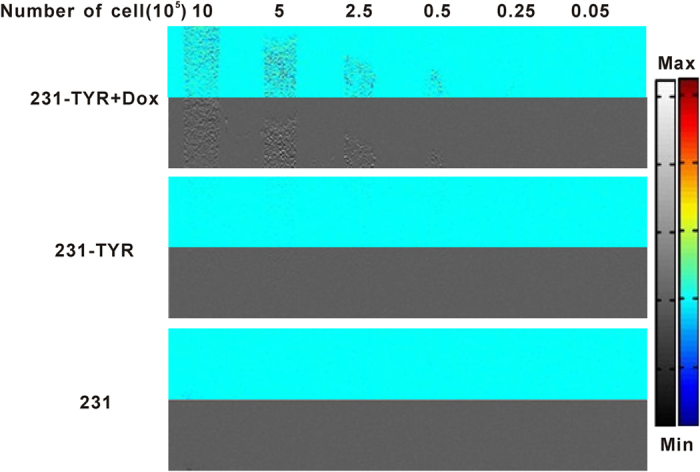
Photoacoustic images of the gel phantom with different concentrations of cells.

Figure 6 shows the photoacoustic signals of different concentrations of cells ranging from 1 × 10^5^ to 2 × 10^7^ /mL. The cell samples were located 2 mm below the surface of the gel phantoms, and the photoacoustic signals could be easily detected in 231-TYR + Dox cells with a cell concentration as low as 1 × 10^6^/mL (5 × 10^4^ 231-TYR + Dox cells). The signal increased with increasing concentration of melanotic cells, while 231-TYR cells without Dox incubation and negative control 231 cells could not produce detectable photoacoustic signal even at 2 × 10^7^/mL.

### Cell uptake study of ^18^F-5-FPN

**Figure 7:**
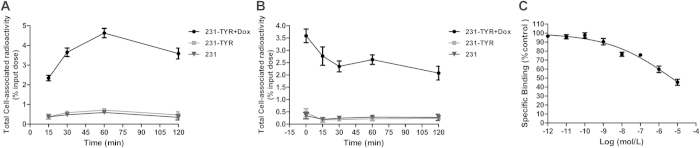
Cell uptake studies of ^18^F-5-FPN. (**A**) Uptake of ^18^F-5-FPN in 231-TYR + Dox, 231-TYR and 231 cells after incubation with ^18^F-5-FPN at 37 °C for 30, 60 and 120 min. All results expressed as percentage of input dose as a result of triplicate measurements ± SD. (**B**) The ^18^F-5-FPN cell uptake efflux study performed in 231-TYR+Dox, 231-TYR and 231 cells at the serial time points. (**C**) competitive cell-binding assay of ^19^F-5-FPN to 231-TYR + Dox cells, all results expressed as percentage of cellular uptake inhibition ratio.

Uptake levels of ^18^F-5-FPN in 231-TYR + Dox, 231-TYR and 231 cells are shown in Fig. 7A. ^18^F-5-FPN quickly accumulated in 231-TYR + Dox cells, with uptake values 4.63 ± 0.24% at 60 min (*n *= 3). In comparison, no or rarely significant accumulation of ^18^F-5-FPN in 231-TYR and 231 cells was observed, with 60 min uptake values of 0.71 ± 0.06% and 0.60 ± 0.05%, respectively. As shown in Fig. 7B, the cell efflux study found that ^18^F-5-FPN efflux from both the 231-TYR + Dox and the contrast cells were most pronounced within the first 30 min; the values were determined to be 2.35% (30 min) for the 231-TYR + Dox cells, 0.18% and 0.24% (30 min) for the 231-TYR and 231 cells, respectively. After 30 min, cell uptake values were maintained with a relatively stable level. Administration of cold ^19^F-5-FPN inhibited the binding of ^18^F-5-FPN in 231-TYR + Dox cells in a concentration-dependent pattern shown in Fig. 7C, illustrating the specificity of binding of ^18^F-5-FPN to melanin *in vitro*.

## Discussion

In this study, we successfully constructed a lentiviral vector complex containing *TYR* as a reporter gene and used the Tet-on system to control its expression. After transducing *TYR* into the breast cancer cell line MDA-MB-231, a stable line expressing *TYR* (231-TYR) was established and screened. We verified that Dox induction could precisely regulate the expression of *TYR*. Further, we demonstrated that tyrosinase, as a multifunctional reporter gene product, could be used for MRI/PET/PAI multimodality imaging *in vitro*.

*TYR* has been used as a reporter gene for magnetic resonance imaging^25^. Most previous studies using *TYR* as a MRI reporter gene have analysed the changes in T1 signal; nonetheless, Fe (III) also has an impact on the T2 relaxation time. In this study, we observed that *TYR* changed the T1 and T2 relaxation times (Fig. 5), consistent with the signal changes observed in images of pigmented melanoma tumours^33^. Quantitative analysis revealed that the T2 relaxation time changed more than that of T1 in 231-TYR + Dox cells. Photoacoustic imaging (PAI) can be used for functional and molecular imaging with endogenous and exogenous contrast agents. Melanin is a common endogenous contrast agent^34^. In our study, photoacoustic signal changes were only detected in melanotic 231-TYR + Dox cells (Fig. 6). Signal detection was very sensitive, identifying signals from only 5 × 10^4^ 231-TYR + Dox cells. The sensitivity in our study was lower than the results of Qin *et al.*^25^, which may be related to difference in instrumentation or different levels of *TYR* expression. We prepared and evaluated ^18^F-5-fluoro-N-(2-[diethylamino]ethyl)picolinamide (^18^F-5-FPN), which has a high affinity with melanin, in our previous study^23^. In this study, ^18^F-5-FPN specifically bound to the melanin in 231-TYR + Dox cells, and blocked with excess nonradioactive standards (Fig. 7), demonstrating the feasibility of *TYR* as a reporter gene for PET imaging.

In the three different imaging modalities, PAI has the highest sensitivity. However, when spatial resolution of 1 mm is necessary, its penetration is less than 5 cm because of the optical attenuation effect. In addition, ultrasound signals cannot penetrate hollow visci or lung tissue owing to the acoustic impedance effect. PET and MRI do not suffer from these limitations. MRI shows a characteristic signal pattern on T1WI and T2WI, with high spatial resolution. ^18^F-5-FPN for PET imaging of melanin/melanoma exhibits high specificity, and can provide functional information. Therefore, a single reporter gene for PAI/MRI/PET multimodality imaging could make up for each modality’s shortcomings.

Effective control of the time and level of gene expression is better than the sustained expression of gene in gene therapy. Sustained expression of exogenous genes or protein may result in adverse effects and receptor downregulation. The Tet-On 3G system consists of three parts including a regulating unit, reaction originals that connect with the *TYR* gene, and inducers. Tetracycline repressor factor (Tet repressor, TetR) and ubiquitin promoter (Ubi) compose the regulating unit; TetR is a repressor protein of Tetracycline inducible promoter (TetIIP). TetIIP, an inducible promoter, mediates expression of *TYR* gene. After TetR inhibition on TetIIP was released using Tet (Dox) combines with TetR, TetIIP will induce *TYR* gene expression. In our study, the *TYR* was only expressed in the presence of Dox in 231-TYR cells using western blot, Masson-Fontana silver staining, and immunofluorescence experiments (Figs 2 and 3). Additional studies of the dosage and differences in length of Dox exposure were conducted (Fig. 4). These results confirmed that the Tet-on system quickly responded to Dox, and it could excite the *TYR* gene expression reversibly, quantitatively and reproducibly.

We demonstrated the potential use of *TYR* for PAI/MRI/PET multimodality imaging *in vitro*. In the future, its potential as an *in vivo* probe for multimodal imaging should be investigated for the following reasons: (1) *TYR* is an endogenous highly biocompatible gene, with the potential for low measurable impact when transfected into amelanotic cells. (2) Dox is an attractive agent for inducing gene expression *in vivo*. (3) *TYR* encodes tyrosinase in the transfected cells, which is the key enzyme for synthesising melanin. Melanin is a polymer and contains multiple binding sites for paramagnetic iron ions, while simultaneously binding benzamide radiopharmaceuticals, making PET/MRI feasible. (4) Used as a multifunctional reporter gene for PAI/MRI/PET imaging, *TYR* may not only solve problems of spatial resolution and sensitivity, but may also enable imaging of microvessels involved in angiogenesis by Doppler photoacoustic tomography.

*TYR* also has potential as a therapeutic agent. Melanin, produced with tyrosine kinase expressed by *TYR*, significantly enhances the absorption of light in the near infrared, which is characterised by low absorption and maximum light penetration in tissues. Stritzker *et al.*^35^ used a near-infrared laser to specifically transfer energy to melanin. The transferred energy converted to thermal energy, which then heated the melanin-producing cells to a high temperature, causing protein denaturation and cell death. In addition, benzamide and its analogues have been labelled with radionuclides to irradiate melanomas. The resulting low transient uptake in the excretory organs has been promising. These data indicate that systemic radionuclide therapy using benzamides for the therapy of pigmented melanoma is of considerable potential^36^,^37^. *TYR* transfection of tumours, causing them to synthesise melanin which is subsequently irradiated by radiolabelled benzamides, may be an effective method of unsealed source therapy.

## Conclusions

We successfully demonstrated that transfected human *TYR* can induce the production of melanin in amelanotic cells, and the gene expression can be accurately regulated by the Tet-on system. A preliminary *in vitro* study suggests that *TYR*, as a single reporter gene, could change T1 and T2 relaxation times on MRI, the signals on PAI, and the accumulation of PET tracer, which suggests its feasibility for multimodality molecular imaging. Further studies *in vivo* are necessary.

## Methods

### Construction of the lentivirus vector complex containing *TYR* and the Tet-on system

cDNA encoding human *TYR* (NM_000372.3) in a pcDNA3.1 vector was kindly provided by Dr. Zhen Cheng of Stanford University. Tet-on 3G system used a GV308 vector (TetIIP-MCS-3FLAG-Ubi-TetR-IRES-Puromycin; 12.4 Kb; Gene Chem Co., Ltd, Shanghai, China). *TYR* DNA was amplified by a polymerase chain reaction (PCR) with primers flanking the *TYR* open reading frame with *BamH*I and *Nhe*I restriction enzyme sequences within the 5′ and 3′ primers, respectively, and it was purified using a gel extraction kit (Qiagen^®^, Tiangen Biotech Co., Ltd. Beijing, China). The purified *TYR* encoding the inserted cDNA and the GV308 vector were both digested with *BamH*I and *Nhe*I restriction enzymes (New England Biolabs, Inc., Ipswich MA, USA) and ligated together with DNA ligase (New England Biolabs). The ligation mixture was used to transform *E*. *coli* DH5a competent cells, which were plated on LB broth plates supplemented with puromycin and shaken for 24 h at 37 °C. Bacterial colonies and plasmid DNA were isolated from the resulting colonies. After the recombinant plasmid was identified by DNA sequencing and double restriction enzyme digestion, plasmid preparation (Maxiprep^®^, Qiagen) was performed, and the concentration of the plasmid was measured. Then, the recombinant plasmid DNA and liposomes were co-transfected into human embryonic kidney 293T cells. We collected the cell supernatant containing the lentiviral particles, concentrated it, and measured the virus titre. The recombinant expression vector was named Lenti-X Tet-on 3G-*TYR* and stored at −80 °C.

### Establishing a stable cell line expressing *TYR*

The human breast cancer MDA-MB-231 cell line (No.Tchu155) was obtained from the Cell Bank of the Chinese Academy of Sciences. MDA-MB-231 cells were grown in Leibovitz’s L-15 medium (L-15^®^; Gibco, Carlsbad CA, USA) supplemented with 10% (v/v) foetal bovine serum (Gibco). To establish a stable cell line expressing TYR, the MDA-MB-231 cells were seeded into 6-well plates at a density of 5 × 10^5^ per well and incubated overnight. We co-transduced these cells with the lentivirus Lenti-X tet-on 3G-*TYR* (multiplicity of infection, MOI = 2) and a transfection enhancer polybrene (Gene Chem Co., Ltd, Shanghai, China), then the cells were replaced into the complete medium 10 h after the transduction. Seventy-two hours after transfection, cells were trypsinised and diluted to a 1000 cells/mL single-cell suspension, and seeded into 96-well plates by the limiting dilution method. After the cells adhered, we observed them carefully under a microscope, choosing and marking those holes observed to contain only 1–2 cells. The next day, these cells were cultured in L-15 medium with 10% FBS containing 1 μg/mL puromycin. The medium was changed every 2–3 days. Cells in dishes grew for several weeks until large cell colonies were visible. Dox (2 μg/mL) was added to each well containing colony cells as an inducer, and carefully observed under light microscopy. The colony with the darkest colour was considered to be capable of producing melanin and termed as 231-TYR cells. This colony was trypsinised from the 96-well plate, cultured and used for subsequent experiments.

### Experimental and control groups

The cells were divided into three groups as follows: (1) 231-TYR cells treated with Dox named as 231-TYR + Dox, which was considered the experimental group; (2) 231-TYR cells with no treatment as one control and named as 231-TYR; (3) MDA-MB-231 cells without any treatment named as 231, which was another control.

### TYR detection by western blot

The cells in the six-well plates were washed twice with cold PBS (0.01 M, pH 7.2) and dissolved in 300 μL radio-immunoprecipitation assay buffer containing protease inhibitors. The lysates were centrifuged at 12000 rpm (68.2 g) for 15 min at 4 °C, and the supernatants collected. Total cellular proteins (20 μg per lane) were resolved using 10% sodium dodecyl sulphate polyacrylamide gel electrophoresis (Bio-Rad^®^, Hercules CA, USA) and were transferred to a nitrocellulose filter membrane (Bio-Rad). The membranes were blocked at 4 °C for 1 h in triethanolamine buffered saline solution (TBST) supplemented with 5% non-fat milk. After a brief rinse, the membranes were incubated overnight at 4 °C in TBST containing 5% bovine serum albumin with the primary antibody diluted in TBST (tyrosinase monoclonal antibody, 1:500), (Sigma Chemical Corporation, St. Louis, MO, USA). A glyceraldehyde-3-phosphate dehydrogenase (GAPDH, 1:1000 Santa Cruz Biotechnology, Santa Cruz CA, USA) polyclonal antibody was used as an internal control protein. The blots were washed three times with TBST for 10 min, followed by 1-h incubation with a horseradish peroxidase-conjugated anti-mouse IgG antibody (1:2000, Santa Cruz) at room temperature. The antigen-antibody peroxidase complex was visualised using enhanced chemiluminescence reagents (ECL^®^, Amersham Biotechnology, Piscataway NJ, USA) according to the manufacturer’s protocol.

### Assessment of cellular tyrosinase activity

The sample preparation procedure was the same as that described in the western blot assay. After quantifying the protein levels, the concentration of samples was adjusted to 0.5 μg/μL. The tyrosinase activity was measured as per the published protocols with some modifications^25^. The experiment was conducted in a 96-well flat-bottom plate. Each well contained 50 μL of cell lysate and 50 μL of 2 mg/mL 3-(3,4-dihydroxyphenyl)-L-alanine (L-DOPA). The mixture was incubated at 37 °C. The absorbance of the reaction mixtures was measured using a plate reader (Omega Bio-tek, Doraville CA, USA) at 475 nm at 1, 2, 3, and 4 h.

### Masson–Fontana silver staining

The three groups of cells were grown overnight in a 6-well plate at a density of 5 × 10^5^ per well with sterilised coverslips. The cells adhering to the coverslips were fixed in iced acetone at 4 °C for 10 min, and then dried at room temperature. The melanin pigment content of these cells was visualised with Masson–Fontana silver staining as per the published protocol^38^. First, the coverslips were rinsed with PBS, then incubated with silver ammonia solution in the dark at 56 °C for 35–40 min. After rinsing in distilled water, the cells were quickly incubated with sodium thiosulfate solution for 1 min. Finally, the coverslips were incubated with neutral red staining solution for 5 min, sealed, and observed under a microscope (Nikon Eclipse 90i; Kawasaki, Kanagawa, Japan). Noticeable black particles could be seen in the 231 TYR + Dox cells.

### Cell immunofluorescence study

The sample preparation procedure was the same as that for the Masson–Fontana silver staining. The coverslips were rinsed with PBS, blocked with 1% bull serum albumin and incubated with a primary antibody (mouse anti-TYR, diluted 1:500; Sigma) overnight at 4 °C. After rinsing in PBS, the cells were incubated with a diluted secondary antibody (Alexa Fluor 488-labelled goat anti-rabbit IgG, diluted 1:200, Beyotime, Beijing, China) at 37 °C for 60 min. Finally, the coverslips were incubated with 4–6-diamidino-2-phenylindole (DAPI; Beyotime) for 5 min, sealed with an agent resistant to quenching, and observed under a confocal microscope (LSM 710: Zeiss, Oberkochen, Germany).

### Measurement of melanin content in 231-TYR cells regulated by Dox

A sample of the 231-TYR cells was digested, re-suspended and cultured in flasks overnight. Then, these cells were incubated with Dox in serial concentrations (10–4000 ng/mL) at 37 °C for 48 h. Melanin content of these cells was measured as described previously with some modifications^39^. The cultured cells were harvested and washed with PBS. They were incubated in 500 μL of 1 N NaOH in an 80 °C water bath for 2 h, then the solution was mixed. After determination of protein content, protein concentration was adjusted to 0.5 μg/μL, and the extracts were then transferred into 96-well plates in triplicate with 50-μL aliquots. The relative melanin content of samples was determined by measuring their absorbance at 405 nm. Results were expressed as absorbance of 405 nm per mg protein.

The incubation time with Dox was investigated to assess for any effect on melanin production in 231-TYR cells. After the cells were cultured in flasks overnight, they were placed in fresh medium containing Dox (2 μg/mL), then continually incubated for 0, 1, 2, 4, 8, 16, 24, 36, 48, and 72 h. Melanin content at different incubation times was measured as described previously.

To assess the impact of Dox on TYR expression, the changes in melanin content after withdrawing Dox at different times were studied in 231-TYR+Dox cells. We cultured the 231-TYR cells with medium containing Dox (2 μg/mL) for 48 h, then replaced the medium with fresh medium without Dox. The cells were digested and collected for determination of melanin content after Dox was removed at 0, 1, 2, 4, 8, 16, 24, 36, and 48 h.

### Cell MRI

Cell phantoms were prepared as follows^25^: the 96-well PCR plates were embedded in a cuboid container filled with 1% UltraPure^TM^ agarose gel (Invitrogen, Carlsbad, CA, USA). After solidification, the tubes were pulled out, and then the bottoms of the resulting holes were filled with 100 μL of 1% agarose. Different concentrations of cells (100 μL, ranging from 2.5 × 10^7^/mL to 1 × 10^8^/mL) suspended in 1% agarose were layered into the middle part of the holes, and then the surface of the phantom was covered with thin 1% agarose gel. MRI was performed using a Bruker 4.7 T/30 cm MRI Imaging System (Bruker Instrument Co., Ltd, Karlsruhe, Germany) with a 72-mm Agilent^®^ radiofrequency (RF) coil. The imaging protocol consisted of axial T1- and T2-weighted fast spin echo (FSE) sequences. T1WI was acquired with the following parameters: repetition time (TR): 350 ms; echo time (TE):11 ms; field of view (FOV): 4.0 × 4.0; matrix size: 256 × 256; slice thickness: 1 mm. T2WI was acquired with the following parameters: repetition time (TR): 2000 ms; echo time (TE): 36 ms; field of view (FOV): 4.0 × 4.0; matrix size: 256 × 256; slice thickness: 1 mm. Image analysis was performed using Paravison operational software.

### Cell PAI

Agarose phantoms were prepared using the PCR tubes. The bottoms of the tubes were filled with 1% agarose gel in distilled water (150 μL). After being cooled down, different concentrations of cells (50 μL) ranging from 1 × 10^5^/mL to 2 × 10^7^/mL suspended in 1% agarose were filled into the middle part of the tubes, then the tops of the tubes were filled with 1% agarose. An acoustic-resolution photoacoustic microscopy system independently manufactured by the National Laboratory for Optoelectronics, Huazhong University of Science and Technology (Wuhan, China) was used to acquire photoacoustic images with a laser at excitation wavelength of 532 nm, a focal depth of 6 mm, pulse width of 6 ns and pulse repetition of 30 Hz.

### Cell uptake studies of ^18^F-5-FPN

Preparation of ^18^F-5-FPN was conducted with the same protocol as described in our previous study^23^. The cellular uptake studies were performed in all experimental and control groups (231-TYR + Dox, 231-TYR, and 231 cells). Cells at a density of 1 × 10^5^ per well were seeded in 24-well plates and incubated overnight. Then, the cells were incubated with 0.2 mL of medium containing 37 kBq (0.5 pM) of ^18^F-5-FPN at 37 °C. At 30, 60, or 120 min after incubation, the medium was removed and cells were washed three times with PBS (pH 7.4) and lysed with 1 N NaOH for 5 min at room temperature. The radioactivity of the cell lysate was measured by a gamma counter (2470, WIZARD; PerkinElmer, Waltham MA, USA). For the cell efflux study, these cells were implanted into plates overnight. ^18^F-5-FPN (37 kBq, 0.5 pM) was added to each properly and incubated for 2 h at 37 °C. After being washed twice with PBS, the cells were incubated in a culture medium for 15, 30, 60 or 120 min. Then, the cells were lysed with 1 N NaOH. For the blocking study, 1 × 10^5^ 231-TYR + Dox cells were seeded overnight and were incubated at 37 °C for 1 h with ^18^F-5-FPN (37 kBq, 0.5 pM) in the presence of 100 μL standards ^19^F-5-FPN (10^−12^ to 10^−5^ M). Then, the cells were washed and the radioactivity measured as with the celluar uptake study.

### Statistical analysis

Quantitative data were expressed as mean ± standard deviation (SD). Means were compared using one-way ANOVA and the Student’s *t*-test with *P* < 0.05 indicating statistical significance.

## Additional Information

**How to cite this article**: Feng, H. *et al.* TYR as a multifunctional reporter gene regulated by the Tet-on system for multimodality imaging: an *in vitro* study. *Sci. Rep.* **5**, 15502; doi: 10.1038/srep15502 (2015).

## Figures and Tables

**Figure 1:**
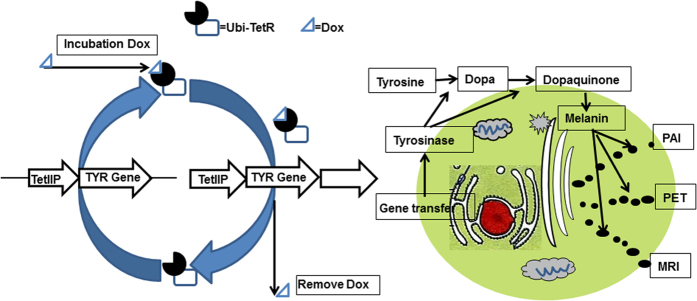
Principle of the Tet-on inducible gene expression system regulating tyrosinase (*TYR*) gene expression using Dox. The *TYR* reporter gene is inserted into cells through the method of gene transfer. Gene expression product tyrosinase, the key enzyme, catalyses the process in melanin production. Melanin then serves as a multifunctional target for photoacoustic imaging (PAI), positron emission tomography (PET) and magnetic resonance imaging (MRI) multimodal imaging.
